# Microbial ecology of a shallow alkaline hydrothermal vent: Strýtan Hydrothermal Field, Eyjafördur, northern Iceland

**DOI:** 10.3389/fmicb.2022.960335

**Published:** 2022-11-17

**Authors:** Katrina I. Twing, L. M. Ward, Zachary K. Kane, Alexa Sanders, Roy Edward Price, H. Lizethe Pendleton, Donato Giovannelli, William J. Brazelton, Shawn E. McGlynn

**Affiliations:** ^1^School of Biological Sciences, The University of Utah, Salt Lake City, UT, United States; ^2^Department of Microbiology, Weber State University, Ogden, UT, United States; ^3^Earth-Life Science Institute, Tokyo Institute of Technology, Tokyo, Japan; ^4^Department of Geosciences, Smith College, Northampton, MA, United States; ^5^School of Marine and Atmospheric Sciences, Stony Brook University, Stony Brook, NY, United States; ^6^Department of Biology, University of Naples “Federico II”, Naples, Italy; ^7^Center for Sustainable Resource Science, RIKEN, Saitama, Japan

**Keywords:** hydrothermal vent, alkaline, Iceland, microbial diversity, 16S rRNA

## Abstract

Strýtan Hydrothermal Field (SHF) is a submarine system located in Eyjafördur in northern Iceland composed of two main vents: Big Strýtan and Arnarnesstrýtan. The vents are shallow, ranging from 16 to 70 m water depth, and vent high pH (up to 10.2), moderate temperature (T_*max*_ ∼70°C), anoxic, fresh fluids elevated in dissolved silica, with slightly elevated concentrations of hydrogen and methane. In contrast to other alkaline hydrothermal vents, SHF is unique because it is hosted in basalt and therefore the high pH is not created by serpentinization. While previous studies have assessed the geology and geochemistry of this site, the microbial diversity of SHF has not been explored in detail. Here we present a microbial diversity survey of the actively venting fluids and chimneys from Big Strýtan and Arnarnesstrýtan, using 16S rRNA gene amplicon sequencing. Community members from the vent fluids are mostly aerobic heterotrophic bacteria; however, within the chimneys oxic, low oxygen, and anoxic habitats could be distinguished, where taxa putatively capable of acetogenesis, sulfur-cycling, and hydrogen metabolism were observed. Very few archaea were observed in the samples. The inhabitants of SHF are more similar to terrestrial hot spring samples than other marine sites. It has been hypothesized that life on Earth (and elsewhere in the solar system) could have originated in an alkaline hydrothermal system, however all other studied alkaline submarine hydrothermal systems to date are fueled by serpentinization. SHF adds to our understandings of hydrothermal vents in relationship to microbial diversity, evolution, and possibly the origin of life.

## Introduction

Alkaline submarine hydrothermal vents have been proposed as potential sites for the origin of life on Earth (and potentially elsewhere in the solar system) because they promote strong proton and redox gradients and facilitate the abiotic synthesis of organic molecules (Russell, 2009; Russell et al., 2010; Price et al., 2017). Specifically, the geochemistry of these systems appears to provide a template for autotrophic metabolic pathways (McCollom and Seewald, 2007; Proskurowski et al., 2008; Hudson et al., 2020), such as the Wood-Ljungdahl pathway shared by methanogens, acetogens, and sulfate-reducers (Martin and Russell, 2007; Adam et al., 2018; Hudson et al., 2020), which may be one of the most ancient metabolic pathways (Ward and Shih, 2019). Previous work on submarine alkaline hydrothermal vents has focused on systems fueled by serpentinization, such as Lost City (Kelley et al., 2005; Lang and Brazelton, 2020; Brazelton et al., 2022), Prony (Monnin et al., 2014; Quéméneur et al., 2014; Postec et al., 2015), and Old City hydrothermal fields (Lecoeuvre et al., 2021). While incredibly valuable, these studies have left many open questions about how opportunities for microbial life may differ in alkaline hydrothermal vents with different geological bases.

**FIGURE 1:**
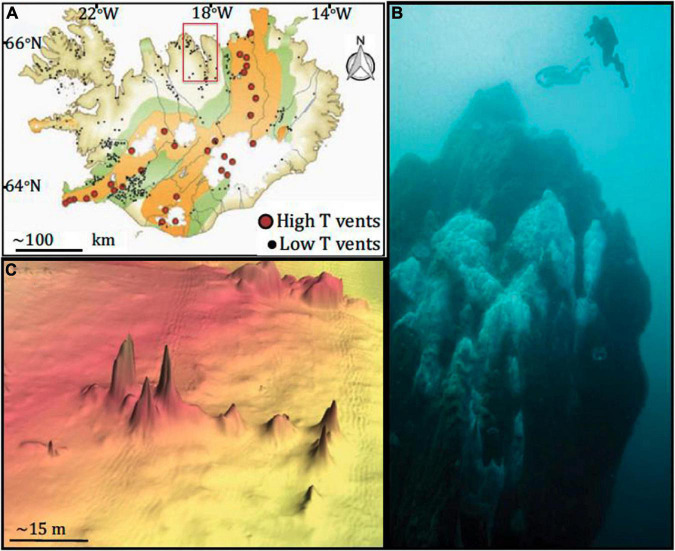
Map of sampling site and vents. **(A)** Map of Iceland, highlighting hydrothermal vents. Eyjafjördur, where Big Strýtan and Arnarnesstrýtan are located, is highlighted by the red box; **(B)** photograph of Big Strýtan chimney (courtesy of E. Bogason); **(C)** bathymetric map of Arnarnesstrýtan. Figure from Price et al. (2017). The figure is reprinted with permission under a Creative Commons CC-BY license.

Strýtan Hydrothermal Field (SHF), while highly alkaline, is not a serpentinizing system, providing a point of comparison to other alkaline sites. Fresh groundwater is heated, enriched in silica, and brought to high pH before venting into the bottom of the marine Eyjafördur. As the hydrothermal and surrounding marine waters mix, they precipitate magnesium silicate minerals which form chimneys rising up to 55 meters from the seafloor (Figure 1) and create steep sodium ion gradients. Harnessing energy as ion gradients across membranes is as universal as the genetic code (Lane and Martin, 2012). As a result, environments with these types of gradients may be important for considering the origin of life (Price et al., 2017); the Na^+^ cell membrane pump is crucial for many ancient microbial lineages, including methanogens and acetogens, and is involved in adenosine triphosphate (ATP) synthesis and carbon assimilation in diverse organisms (Lane and Martin, 2012; Branscomb et al., 2017). Although not a focus of this study, it is fascinating to consider an energy source invoked in origin of life theory as possibly being operative in today’s microbial communities.

Very little microbiological research has been conducted at SHF. In a study from 2001, researchers exploring vent fluids isolated Aquificales and Korarchaeota from clone libraries (Marteinsson et al., 2001b). However, a full census of microbial diversity at SHF has not been previously conducted. By characterizing the microbial diversity of vent fluids and mineral precipitates at SHF, we can better understand the ability of alkaline hydrothermal systems, and this unique site in particular, to support microbial communities and better constrain their potential roles in the origin of life.

## Materials and methods

### Sample collection

In August 2017, we performed a 7-day field-sampling campaign to SHF, where scientific SCUBA divers collected actively venting fluids from both Big Strýtan and Arnarnesstrýtan using 30 m of cleaned polypropylene tubing by swimming to the appropriate sampling location and holding the tubing inlet in the actively venting water. On the boat, the tubing was connected to two 12V inline electric galley pumps to pull water from the vent. During sample collection, pH and temperature were monitored to ensure the capture of vent fluids, rather than background seawater. Vent fluid samples for DNA analyses were collected in triple-rinsed 20 L carboys and background seawater samples were collected in sterilized 4 L carboys. A field station laboratory was established in the port dive shop, where collected fluid samples were filtered through 0.22 μm Sterivex cartridge filters (Millipore, MA, United States) using a peristaltic pump and flash-frozen in liquid N_2_.

Hydrothermal vent chimney samples from Big Strýtan and Arnarnesstrýtan were collected by scientific divers by breaking off small (∼15 cm × 5 cm) pieces into sterile Whirl-pak bags (Nasco, WI, United States). Back in the field-station laboratory, the chimney samples were subsampled into distinct chimney layers (Supplementary Figures 1, 2) using a flame-sterilized scalpel, and these solid samples were immediately frozen in liquid N_2_. All frozen filter and chimney samples were shipped back to the University of Utah in dry shippers and stored at –80°C until nucleic acid extraction.

### DNA extraction

DNA was extracted from the Sterivex filter cartridges using a lab protocol previously developed for low-biomass alkaline fluid samples (Brazelton et al., 2022). Briefly, cell lysis was performed by alternating freeze/thaw cycles in an alkaline buffer, followed by purification with phenol-chloroform and precipitation with ethanol. Additionally, a laboratory extraction blank (ICEd062, Supplementary Table 1), consisting of a new Sterivex filter, was extracted alongside the fluid samples to track any potential laboratory contamination.

DNA was extracted from ∼0.5 to 1.0 g of homogenized chimney samples using the MP Biomedical FastDNA Spin kit (CA, United States), per the manufacturer’s instructions, with the exception of the use of a Mini-Beadbeater-16 (Biospec Products, OK, United States) in place of the recommended FastPrep Instrument. Technical replicates were pooled, to a total starting mass of 1.5–3.5 g per chimney sample, using an Amicon Ultra-2 Centrifugal Filter Unit with Ultracel-30 membrane (Millipore, Darmstadt, Germany). All DNA samples were purified using AMPure XP speedbeads (Rohland and Reich, 2012) and quantified via the Qubit high-sensitivity DNA assay (Invitrogen, CA, United States). A list of all DNA samples included in the study can be found in Supplementary Table 1.

### 16S rRNA sequencing and data analysis

DNA samples were submitted to the Michigan State University Research and Technology Support Facility Genomics Core for sequencing of the V4 region of the 16S rRNA gene on an Illumina MiSeq platform using the universal duel-indexed Illumina fusion primers 515F-806R (Kozich et al., 2013). Amplicon concentrations were normalized and pooled using an Invitrogen SequalPrep DNA Normalization Plate. After library quality control and quantitation, the pool was loaded on an Illumina MiSeq v2 flow cell and sequenced using a standard 500 cycle reagent kit. Base calling was performed by Illumina Real Time Analysis (RTA) software v1.18.54. Output of RTA was demultiplexed and converted to fastq files using Illumina Bcl2fastq v1.8.4.

16S rRNA gene amplicon sequences were processed with cutadapt v. 1.15 (Martin, 2011) and DADA2 v. 1.10.1 (Callahan et al., 2016), including quality trimming and filtering of reads, removal of chimeras, and inference of amplicon sequence variants (ASVs). Taxonomic classification of all ASVs was performed with DADA2 using the SILVA reference alignment (SSURef v138) and taxonomy outline (Pruesse et al., 2012) with the default minimum bootstrap value of 50 for taxonomic assignments (Callahan et al., 2016).

Quality control of sequence data was performed by removing putative contaminant sequences from the dataset. For vent fluid and background seawater samples, which were collected by filtration through Sterivex filters, a laboratory extraction blank (ICEd062; Supplementary Table 1) was sequenced and the 73 ASVs detected in that extraction blank were removed from the 8 vent fluid samples and 2 background seawater samples. Chimney samples were extracted via a DNA extraction kit, and 203 ASVs belonging to the genera outlined in Sheik et al. (2018) as putative kit contaminants were removed from the 12 chimney samples. A detailed list of all taxa removed from the dataset can be found in Supplementary Table 2. Additionally, any sequences classified to Domain “NA” or “Eukaryota” and all Bacteria classified as “Chloroplast” or “Mitochondria” were removed from the dataset.

After removing potential contaminant sequences, raw counts were converted to proportions to normalize for variations in sequencing depth among samples. The proportional abundances of all 872 unique ASVs among all seawater, vent fluid, and chimney samples were used to calculate the Morisita-Horn community dissimilarity between each pair of samples. Similar results were obtained with other metrics of dissimilarity (e.g., Bray-Curtis, Sørensen). The multi-dimensional scaling (MDS) plot was generated from a table of ASV proportional abundances across all sample categories (vent fluids, chimneys, and background seawater) using the distance, ordinate, and plot_ordination commands in the R package phyloseq v.1.26.1 (McMurdie and Holmes, 2013). All sequence data is available in the NCBI Sequence Read Archive (SRA) under BioProject PRJNA861241.

### Comparison to other high pH vents

We cross-referenced the taxa detected in previous studies of alkaline submarine vents (Quéméneur et al., 2014; Postec et al., 2015; Lecoeuvre et al., 2021; Brazelton et al., 2022) and alkaline hot springs (Reysenbach et al., 1994; Marteinsson et al., 2001a; Blank et al., 2002; Nakagawa and Fukui, 2003; Purcell et al., 2007; Boomer et al., 2009; Meyer-Dombard et al., 2011; Tobler and Benning, 2011; Meyer-Dombard and Amend, 2014; López-López et al., 2015; Martinez et al., 2019) with the abundant (>1% of any sample) taxa detected in this study (Supplementary Table 3). For studies conducted with clone-libraries, we included all reported taxa (Reysenbach et al., 1994; Marteinsson et al., 2001a; Blank et al., 2002; Nakagawa and Fukui, 2003; Purcell et al., 2007; Boomer et al., 2009; Meyer-Dombard et al., 2011; Tobler and Benning, 2011; Meyer-Dombard and Amend, 2014; Quéméneur et al., 2014; Postec et al., 2015), while for studies conducted with amplicon and/or metagenomic sequencing, we included taxa representing a relative abundance of >1% of any sample (López-López et al., 2015; Martinez et al., 2019; Lecoeuvre et al., 2021; Brazelton et al., 2022). Similarities between phylum through genus for Archaeon and class through genus for Bacteria were included with the lowest common taxonomic level reported in Supplementary Table 3.

Additionally, the only previous microbiological study of SHF was published in 2001 (Marteinsson et al., 2001b) using Sanger sequencing of clone libraries. Given that microbial taxonomy is ever evolving, in order to compare the taxonomy of these previously detected microorganisms to our current data, we classified the sequences reported in Marteinsson et al. (2001a) by searching them against the current (November 2021) NCBI nr database using the BLASTn function (Altschul et al., 1990). Data originally reported in Marteinsson et al. (2001a) and the updated taxonomic assignments can be found in Supplementary Table 4.

### Aqueous geochemistry

Fluid samples were collected directly from the tubes through which microbial samples were taken. Temperature was measured *in situ* with a Thermo Fisher Scientific temperature probe in an underwater housing constructed at the Max Planck Institute for Marine Microbiology. pH was measured in the field just after surfacing from each dive using a Hanna HALO Bluetooth pH meter with temperature compensation. Samples for major anion concentrations were preserved in the field by 0.2 μm syringe filtered into acid washed HDPE Nalgene^®^ bottles and frozen until analysis. Analyses were performed in the Wehrmann Biogeochemistry Laboratory, School of Marine and Atmospheric Sciences, Stony Brook University using a Metrohm 930 compact ion chromatograph with matrix elimination. Samples for major cation concentrations were preserved in the field by 0.2 μm syringe filtered into acid washed HDPE Nalgene^®^ bottles, acidified to 0.1% acid with ultrapure nitric acid, and frozen until analysis. Analyses were performed in the Black Paleooceanography Laboratory, School of Marine and Atmospheric Sciences, Stony Brook University using a Horiba Ultima 2C inductively coupled plasma–optical emission spectrometer (ICP-OES).

## Results

### Geochemistry of Strýtan Hydrothermal Field

Strýtan Hydrothermal Field is located in Eyjafördur in northern Iceland and consists of two main vent sites: Big Strýtan and Arnarnesstrýtan (Figure 1). Each site consists of several large (up to 55 m tall) chimneys in shallow water (<60 m depth) composed of saponite, a magnesium silicate material with basaltic host rock (Geptner et al., 2002; Price et al., 2017). The chimneys vent pH 10.2 freshwater that is enriched in dissolved silicate and slightly elevated in H_2_ (0.1–5.2 μM) and methane (0.5–1.4 μM) (Price et al., 2017). In contrast to other alkaline hydrothermal vents, the Strýtan Hydrothermal Field is hosted in basalt and therefore the high pH is not created by serpentinization but rather a combination of (1) plagioclase hydrolysis, coupled with calcite precipitation, and (2) hydration of Mg in pyroxene and olivine in basalt (Geptner et al., 2002; Price et al., 2017). SHF vent fluids are sourced in groundwater, and the steep sodium ion gradients from the fluids at SHF (from <3 to 468 mM), may be important for understanding processes involved in the origin of life (Lane and Martin, 2012; Price et al., 2017).

**FIGURE 2:**
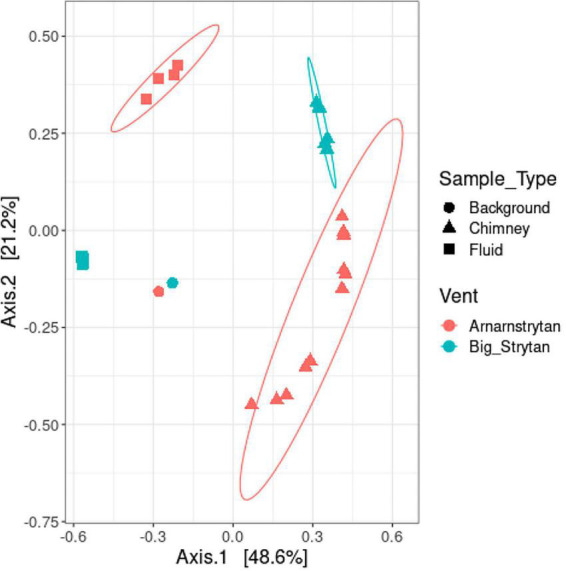
Distinct microbial communities between sample types (background seawater, chimney, and vent fluids) and different vents (Big Strýtan, pink; Arnarnesstrýtan, teal). Multi-dimensional scaling plot of community dissimilarity, calculated with the Morisita-Horn dissimilarity index.

Five types of samples were collected during our expedition: (1) vent fluids from Big Strýtan vent, (2) chimney samples from Big Strýtan vent, (3) vent fluids from Arnarnesstrýtan vent, (4) chimney samples from Arnarnesstrýtan vent, and (5) background seawater samples, each of which harbored distinct microbial communities (Figure 2). The chimney samples from the two vents, while distinct from one another, were more similar to each other than to any of the fluid samples and all the vent and chimney samples were distinct from the background seawater (Figure 2).

**FIGURE 3:**
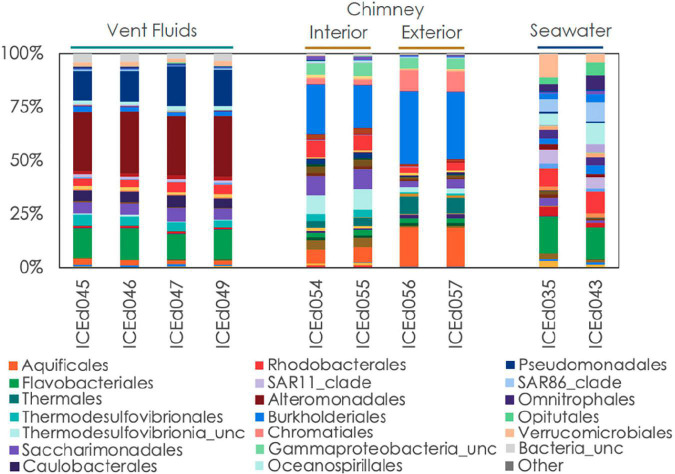
Relative abundance of microbial communities in Big Strýtan vent fluids, chimneys, and background seawater at the order level.

The background seawater sample exhibited chemical characteristics typical of seawater (e.g., pH 8, mM concentrations of ions; Table 1; Pinet, 2019). The chemistry of the vent fluids from Big Strýtan and Arnarnesstrýtan were similar, with high pH (∼10) and generally low in dissolved ions. This is in agreement with previous studies (Marteinsson et al., 2001b; Price et al., 2017). The sample from Arnarnesstrýtan had slightly higher dissolved ions, likely as a result of mixing with seawater, either deeper in the hydrothermal edifice or during sampling. Of particular interest, H_2_S and Fe were slightly elevated in the vent fluids relative to seawater. While the relative contributions to the H_2_S content of microbial SO_4_^2–^ reduction and mobilization from subsurface basalts during water-rock reactions has not been confirmed, the abundance of known dissimilatory sulfate reducing microorganisms in the vent fluids (such as Thermodesulfovibrionales, e.g., Sekiguchi et al., 2008; Figure 3) does suggests a role for biological sulfur cycling.

**TABLE 1:** Geochemical parameters measured at Strýtan hydrothermal field.

	Background seawater	Big Strýtan	Arnarnesstrýtan
Date	28 August 2017	31 August 2017	27 August 2017
pH	8.0	10.2	9.4
SO_4_ (mM)	27.3	0.1	1.2
H_2_S (μ**M)**	n.d.	28.0	10.9
Cl (mM)	530.0	1.3	21.6
Br (μ**M)**	795.3	2.3	36.5
Na (mM)	412.4	4.1	15.2
Ca (mM)	9.9	0.08	0.82
K (mM)	9.12	0.04	0.25
Mg (mM)	49.5	0.1	1.0
Si (mM)	0	1.5	1.3
Fe (μ**M)**	n.d.	1.8	0.32
Sr (μ**M)**	86.0	0.4	3.1

n.d., no data.

### Microbial community of Big Strýtan vent

The microbial communities between background seawater, vent fluids, and chimney material from the Big Strýtan site are distinct (Figure 3). Vent fluids from Big Strýtan were dominated by members of the orders Alteromonadales (29.6%), Pseudomonadales (19.1%), Flavobacteriales (14.9%), Saccharimonadales (6.4%), Caulobacterales (6.0%), and Thermodesulfovibrionales (5%; Figure 3). Two of the three most abundant ASVs in Big Strýtan vent fluids both belonged to the genus *Pseudoalteromonas* within the class Alteromonadales. The best hit for ASV sq4, which made up 18–21% of the vent fluid community, was *Pseudoalteromonas carrageenovora*, isolated from extremely deep marine sediments from the Challenger Deep (Chen et al., 2021; Table 2). ASV sq11, classified as *Pseudoalteromonas* and comprising 5–7% of the vent fluids, had a best hit from a recently formed, circumneutral hydrothermal vent (García-Davis et al., 2021; Table 2). There were five ASVs belonging to the Pseudomonadales in Big Strýtan vent fluids (Supplementary Table 3). The third most abundant ASV in Big Strýtan vent fluids was sq31 (7.8%), which was classified as *Acinetobacter* sp. and has 100% similarity to a sequence from a subsurface aquifer (Table 2; Espín et al., 2020). Two other ASVs belonging to the Pseudomonadales were classified at least the genus level and their best hits were also from hydrothermal vents: ASV sq63 (3.3%) is 100% similar to *Psychrobacter* sp. found at the recently formed vent referenced above (García-Davis et al., 2021) and ASV sq113 (2.2%) is 99.6% similar to *Acinetobacter johnsonii* (unpublished; Supplementary Table 3). The two ASVs of *Flavobacterium* spp. had best hits that originated in Arctic sediments (*F. petrolei*, Chaudhary et al., 2019) or volcanic soil samples (*F. degerlachei*, Zeglin et al., 2016; Supplementary Table 3). Two ASVs (sq28, sq112) classified as *Thermodesulfovibrio* were close matches to sequences found in terrestrial alkaline hot spring samples from Iceland (Marteinsson et al., 2001a). While the orders Saccharimonadales and Caulobacterales made up 6.4 and 6.0% of the vent fluids, respectively (Figure 3), no individual ASV belonging to these orders was >1% of any sample.

**TABLE 2:** Abundant (>5%) ASVs in big Strýtan vent fluid and chimney samples.

ASV	Best hit			Max abundance (%)			
		
	% Identity	Environment	Accession number	Fluid	Chimney		Background seawater
						
					Interior	Exterior	
sq4_Pseudoalteromonas	100	Deep-sea sediments	MW926624 ^1^	21	0.2	0.01	0.2
sq11_ Pseudoalteromonas	100	Hydrothermal vent	MW826701 ^2^	7.3	0.1	0.002	0.1
sq31_Acinetobacter	100	Subsurface aquifer	MT941733 ^3^	7.8	0.08	0	0.01
sq2_Sutterellaceae	99.6	Hot spring	LK936281 ^4^	0.07	16.6	25.6	0.01
sq3_Thermocrinis	98.8	Hot spring, Iceland	AF361217 ^5^	0.07	6.3	17.4	0
sq16_Thermodesulfovibrionia	95.7	Hot spring	FJ206245 ^6^	0	6.7	2.5	0
sq17_Candidatus_Thiobios	99.6	Marine sediments	FM242256 ^7^	0.03	2.2	9.6	0

^1^Chen et al. (2021). ^2^García-Davis et al. (2021). ^3^Espín et al. (2020). ^4^Anda et al. (2014). ^5^Marteinsson et al. (2001b). ^6^Boomer et al. (2009). ^7^Païssé et al. (2010).

We subsampled a piece of chimney from Big Strýtan into interior and exterior sections for microbial analyses (Supplementary Figure 1). The interior of the Big Strýtan chimney samples, which were in direct contact with actively venting fluids, were dominated by members of the Burkholderiales (22.1%), Thermodesulfovibrionales (9.1%), Saccharimonadales (8.9%), Rhodobacteriales (7.4%), and Aquificales (6.8%) (Figure 3). The exterior of the chimney samples, which were in contact with surrounding seawater, were dominated by Burkholderiales (33.4%), Aquificales (18.2%), and Chromatiales (9.7%) (Figure 3). The most abundant ASV was sq2_Sutterellaceae (Burkholderiales), which accounted for 16.6% of the inner layers and 25.6% of the outer layers of the chimney (Table 2) and had a best hit match from a circumneutral hot spring in Hungary (Anda et al., 2014). ASV sq3, identified as *Thermocrinis albus* (Aquificales) and first isolated from Hvergerthi in Iceland (Eder and Huber, 2002), was 6.3% and 17.4% of the interior and exterior of Big Strýtan chimney samples, respectively. ASV sq16 was classified to the class Thermodesulfovibrionia and was only 95.7% similar to the best hit match in the NCBI SRA database, which was a sequence from an alkaline spring at Yellowstone National Park (Boomer et al., 2009; Table 2). Finally, ASV sq17, comprising 9.6% of the exterior chimney, was classified as Candidatus *Thiobios* (Chromatiales) and had a best hit to a sequence from marine sediments (Table 2).

**FIGURE 4:**
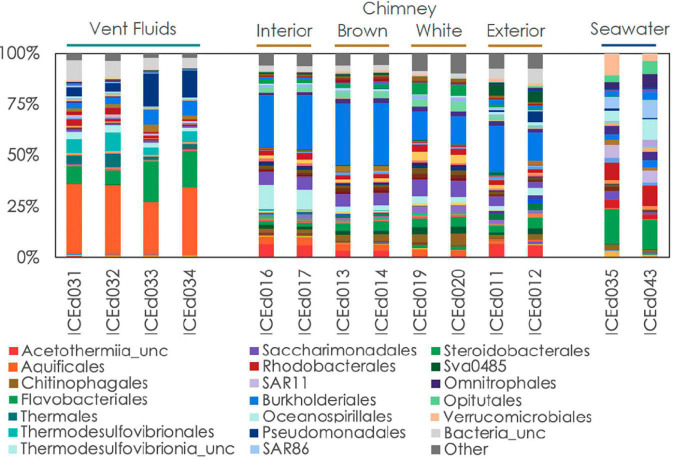
Relative abundance of microbial communities in Arnarnesstrýtan vent fluids, chimneys, and seawater at the order level.

The background seawater samples, which were collected on two separate dives (Supplementary Table 1) shared marked similarities in their community composition and were dominated by members of the Flavobacteriales (17.4%), Rhodobacterales (10.4%), Verrucomicrobiales (10.7%), Oceanospirillales (10.3%), SAR86 (9.5%), Opitutales (6.6%), Omnitrophales (6.6%), and SAR11 (6.5%) (Figures 3, 4). It is important to note that the genera within these orders were distinct from members found in the vent or chimney samples (Supplementary Table 3).

### Microbial community of Arnarnesstrýtan vent

Even at the order level of taxonomy, distinct communities were observed in Arnarnesstrýtan vent fluids, chimney samples, and background seawater (Figure 4). Vent fluids from Arnarnesstrýtan were dominated by members of the orders Aquificales (33.9%), Flavobacteriales (19.5%), Pseudomonadales (16.3%), Thermodesulfovibrionales (9%), Burkholderiales (7.5%), and Thermales (6.4%), as well as a significant proportion of unclassified Bacteria (9.7%) (Figure 4). There were six ASVs belonging to the Aquificales, two of which were “uncultured” *Thermocrinis* sp. and four classified as *Thermocrinis albus* (Table 3). The best hits of all six sequences were from previous studies conducted in alkaline Icelandic hot springs (Marteinsson et al., 2001a; Wirth et al., 2010; Tobler and Benning, 2011), though not specifically at SHF. Three ASVs of *Flavobacterium* spp. all had best hits that originated in Arctic sediments (*F. petrolei*, Chaudhary et al., 2019) or thawing permafrost samples (*F. psychrolimnae*, Aszalós et al., 2020 and *F. degerlachei*, Zeglin et al., 2016) (Table 3 and Supplementary Table 3). *Pseudomonas extremaustralis* (sq55) was also detected in Arnarnesstrýtan vent fluids and has previously been observed in other alkaline hot springs (Table 3; Wang and Pecoraro, 2021). Furthermore, this ASV (Sq55_Pseduomonas) was not detected in background seawater samples. There were two ASVs of *Thermodesulfovibrio* (sq28, sq60), both of which were previously detected in alkaline hot springs in Iceland (Marteinsson et al., 2001a; Supplementary Table 3). ASV sq14 was classified as *Thermus islandicus* and was 100% similar to the type strain, isolated from a hot spring in southern Iceland (Bjornsdottir et al., 2009).

**TABLE 3:** Abundant (>5%) in Arnarnesstrýtan vent fluid and chimney samples.

ASV	Best hit			Max abundance (%)			
		
	% Identity	Environment	Accession number	Fluid	Chimney		Background seawater
						
					Interior	Exterior	
sq3_Thermocrinis	98.8	Hot spring, Iceland	AF361217 ^1^	7.9	0.2	0.8	0
sq15_Thermocrinis	99.6	Hot spring, Iceland	GU233821 ^2^	8.9	0.007	0.009	0
sq20_Thermocrinis	99.2	Hot spring, Iceland	AF361217 ^1^	8.7	0.007	0.006	0
sq14_Thermus	100	Hot spring, Iceland	NR_116442 ^3^	6.3	0.3	0	0
sq18_Flavobacterium	100	Arctic sediment	NR_169457 ^4^	12.5	0	0	0.02
sq55_Pseudomonas	100	Alkaline hot spring	MZ497311 ^5^	5.4	0	0	0
sq2_Sutterellaceae	99.6	Hot spring	LK936281 ^6^	0.6	24.0	2.5	0.01
sq7_Hydrogenophilaceae	100	Microbialites	JQ766812 ^7^	0	6.7	8.0	0
sq13_Thermodesulfovibrionia	99.2	Hot spring	LC422496 ^8^	0.002	7.9	0.1	0
sq39_Rhodocyclaceae	99.6	Deep gold mine	EF205275 ^9^	0.004	1.6	9.6	0
sq41_Acetothermiia	100	Hot spring, Iceland	GU233848 ^2^	0.3	1.8	5.9	0
sq69_Sva0485	99.2	Microbialites	KP589170 ^10^	0	0.8	6.1	0

^1^Marteinsson et al. (2001b). ^2^Tobler and Benning (2011). ^3^Bjornsdottir et al. (2009). ^4^Chaudhary et al. (2019). ^5^Wang and Pecoraro (2021). ^6^Anda et al. (2014). ^7^Couradeau et al. (2017). ^8^Shiraishi et al. (2019). ^9^Onstott et al. (2009). ^10^Corman et al. (2016).

The Arnarnesstrýtan chimney exhibited distinct color zones and was subsampled to assess the microbial diversity of each zone independently (Supplementary Figure 2). The interior-most zone (gray) was in direct contact with venting fluids and expected to be a more anoxic environment than the exterior-most zone (yellow), which was in direct contact with the surrounding seawater (Supplementary Figure 2). The interior Arnarnesstrýtan chimney samples were dominated by members of the Burkholderiales (25.9%), Thermodesulfovibrionia (11.6%), Acetothermiia (6.5%), and Saccharimonadales (5.8%) (Figure 4). Meanwhile, the exterior Arnarnesstrýtan chimney samples were dominated by Burkholderiales (13.7%), Acetothermiia (6.5%), Sva0485 (5.4%), and Pseudomonadales (5.0%) (Figure 4). Similar to the vent fluids, there was a significant proportion of unclassified Bacteria (7%) found in Arnarnesstrýtan chimney samples (Figure 4). Five ASVs from the class Burkholderiales were detected in the chimney samples. The most abundant was ASV sq2_Sutterellaceae, which accounted for 10–20% of the inner layers and 2–3% of the outer layers of the chimney (Table 3). ASV sq66 was identified as *Hydrogenophaga* and was abundant in the exterior layers (white, yellow; Supplementary Table 3). ASV sq7_Hydrogenophilaceae was detected at high abundance (4–7%) in all layers of the chimney sample (Table 3). *Thermodesulfovibrio* spp. were detected in Arnarnesstrýtan vent fluids and in the inner most chimney sample there were three ASVs of unclassified Thermodesulfovibrionia (same Class) that accounted for 7–10% of the replicate samples (Figure 4 and Table 3). Two of those ASVs (sq16, sq134) had sequence similarity of 95% with their closest BLAST hits, both of which were from an alkaline hot spring in Yellowstone National Park (Boomer et al., 2009; Supplementary Table 3). There were two ASVs identified as uncultured Acetothermiia. ASV sq32 was most abundant (4.5%) in the interior layer, while ASV sq41, the closest BLAST hit of which is also from alkaline vents in Iceland (Tobler and Benning, 2011), was abundant (5.9%) in the exterior layer of the chimney (Table 3 and Supplementary Table 3). Finally, an ASV classified as Sva0485 (phylum) was 6% of the exterior (yellow) layer and found in other studies looking at microbialite formation (Table 3; Corman et al., 2016; Couradeau et al., 2017). While the orders Saccharimonadales and Pseudomonadales made up 5.8 and 5.0% of the chimney samples, respectively (Figure 4), no individual ASV belonging to these orders was >1% of any sample. Only one ASV belonging to the domain Archaea accounted for >1% of any sample in the study. This was ASV sq175_Candidatus *Nitrosopumilus*, present in exterior Arnarnesstrýtan chimneys (Supplementary Table 3).

## Discussion

### Microbial ecology of Strýtan Hydrothermal Field

The vent fluids from both Big Strýtan and Arnarnesstrýtan contain an abundance of *Flavobacterium*, *Pseudoalteromonas*, *Psychrobacter*, *Acinetobacter*, and *Pseudomonas* species (Tables 2, 3 and Supplementary Table 3). Type strain members of these genera are all aerobic, heterotrophic bacteria (Akagawa-Matsushita et al., 1992; Bozal et al., 2003; Van Trappen et al., 2004, 2005; Chaudhary et al., 2019), with the exception of *Pseudomonas extremaustralis*, which is capable of nitrate-reduction (López et al., 2009, 2017). Interestingly, almost all of the species detected exclusively in vent fluids are also found in Arctic and Antarctic samples (Bozal et al., 2003; Van Trappen et al., 2004, 2005; López et al., 2009; Chaudhary et al., 2019). None of these ASVs were detected in background seawater samples, so it is unlikely that the samples were “contaminated” by seawater. Instead, the vent fluid samples from Big Strýtan and Arnarnesstrýtan represent a mixing zone within the chimney, where deeply sourced hydrothermal fluids, enriched in H_2_, CH_4_, and H_2_S, come into contact with oxygenated seawater, creating a niche habitat for aerobic to microaerophilic organisms capable of H_2_ and H_2_S-oxidation.

Two ASVs were abundant in both vent fluids from Arnarnesstrýtan and exterior chimney samples from Big Strýtan, but not detected in the other samples. These belonged to *Thermus islandicus* (sq14) and *Thermocrinis albus* (sq3; Tables 2, 3 and Supplementary Table 3). Additional ASVs of *Thermocrinis albus* (sq15, sq20) were detected only in Arnarnesstrýtan vent fluids (Supplementary Table 3). The best hit matches for these four ASVs all originally come from alkaline geothermal vents in Iceland (Marteinsson et al., 2001a; Tobler and Benning, 2011). *Thermus islandicus* is a microaerophilic, mixotrophic, sulfur-oxidizer, originally isolated from a pH 6 hot spring in southern Iceland (Bjornsdottir et al., 2009). *Thermocrinis albus* belongs to the phylum Aquificota and is a microaerophilic chemolithoautotroph that uses the reverse-TCA cycle and is able to utilize H_2_, S^0^, and thiosulfate as electron donors (Eder and Huber, 2002; Hügler et al., 2007; Wirth et al., 2010). These microaerophilic, sulfur-oxidizing organisms, are likely inhabiting an oxic/anoxic mixing zone within Arnarnesstrýtan fluids and a low-oxygen cavity within the exterior of the Big Strýtan chimney. The chemistry of the fluids suggest that seawater was entrained slightly more for Arnarnesstrýtan compared to Big Strýtan. It is possible that this occurred prior to sampling, deeper within the chimney edifice. If true, then the entire edifice might have elevated O_2_ concentrations from seawater, which would help explain the presence of more aerobic metabolisms in Arnarnesstrýtan fluids compared to Big Strýtan.

Taxa with putatively diverse metabolisms were detected within the chimney samples from both vents. Interior chimney samples from both Big Strýtan and Arnarnesstrýtan had an abundance of Thermodesulfovibrionia (sq13, sq16, sq84, sq110, sq117, and sq134) and Sutterellaceae (sq2, sq38, sq78, sq87, sq92, and sq146; Tables 2, 3 and Supplementary Table 3). Members of both these groups are anaerobes and Thermodesulfovibrionia are capable of nitrate- and sulfate-reduction (Morotomi, 2014; Arshad et al., 2017; Umezawa et al., 2021). The exterior chimney samples from both vents contained Acetothermia (sq41; Table 3 and Supplementary Table 3), a phylum that is also referred to OP1 and Bipolaricaulota in the literature. Genomes of Acetothermia contain the complete Wood-Ljungdahl pathway, allowing for potential acetogenesis in these samples (Youssef et al., 2019). Various taxa putatively capable of carbon-fixation *via* the Calvin-Bassam-Benson cycle were detected in exterior samples from Big Strýtan (Ca. *Thiobios*) and Arnarnesstrýtan (Hydrogenophilaceae, *Hydrogenophaga*) chimney (Tables 2, 3 and Supplementary Table 3; Willems et al., 1989; Rinke et al., 2009; Orlygsson and Kristjansson, 2014). The apparent transition from Wood-Ljungdahl dominated autotrophy in interior samples to CBB-driven carbon fixation in the exterior may reflect the different sensitivities of these pathways to O_2_ concentration (e.g., Berg, 2011) across the chimney/seawater boundary. Additionally, the sulfate-reducing Ca. *Desulforudis* (sq149) was detected in exterior Arnarnesstrýtan chimney samples (Chivian et al., 2008). This organism has previously been shown to be capable of carbon fixation *via* the O_2_-sensitive Wood-Ljungdahl pathway (Karnachuk et al., 2019), suggesting low-oxygen conditions extended into at least some distal regions of the chimney interior. Interestingly, no phototrophic bacteria were detected, even in exterior chimney samples, despite the shallow water depth.

Our subsampling of the chimney samples (Supplementary Figures 1, 2) have allowed for us to distinguish oxic, low oxygen, and anoxic habitats within the chimneys. The inferred metabolisms suggest that O_2_ and perhaps SO_4_^2–^ are important electron acceptors, along with NO_3_^2–^, whereas H_2_ and H_2_S may be the most important electron donors. It appears that sulfur metabolisms may play a crucial role. With the observation that O_2_ and SO_4_^2–^ may be entrained deeper within the system, H_2_S observed in the vent fluids may be produced by SO_4_ (or thiosulfate) reduction with H_2_. Support for this observation comes from Rucker et al. (2022), who conducted thermodynamic calculations of Gibbs free energy to evaluate the highest energy yielding reactions as a proxy for microbial metabolism. The most energetically favorable reactions in all mixing schemes for Strýtan includes CO_2_ or O_2_ reduction with H_2_, or O_2_ reduction with H_2_S oxidation. Numerous NO_3_^–^ reduction reactions are also favorable, and NO_3_^–^ reduction with H_2_S oxidation is among the “top five most” energetically yielding reactions.

As noted above, the only previous microbiological study conducted at SHF was published in 2001 and used cultivation-dependent and clone library methodologies (Marteinsson et al., 2001b). Of the ∼100 bacteria described in Marteinsson et al. (2001b), accession numbers were only available for eight of them. To compare the taxonomy of those eight sequences from 2001 to our current dataset, we performed a BLASTn search of the sequences against the current (Nov. 2021) NCBI SRA database (Supplementary Table 4). Most notable is the presence of *Thermocrinis albus* (referred to as Aquificales in Marteinsson et al., 2001b), which dominated clone-libraries in the 2001 study and accounted for 19 and 34% of the amplicon libraries from Big Strýtan chimney and Arnarnesstrýtan vent fluids, respectively, in the present study (Supplementary Table 3, 4). We similarly detected members of the Actinobacteriota, Nitrospirota, Caulobacteraceae, and Burkholderiales at SHF (Marteinsson et al., 2001b).

It should be noted that 16S rRNA derived discussion of metabolic capacity may not accurately represent what is happening in the natural environment. Future study of metagenomic and metranscriptomic data from this site will help clarify the metabolic strategies employed at SHF and the current work is a first insight.

### Comparison of Strýtan Hydrothermal Field to other alkaline hydrothermal vent sites

To put the unique nature of Strýtan Hydrothermal Field into context, we compared the microbial composition reported here to the two most similar ecosystem classes–alkaline submarine hydrothermal vents and alkaline terrestrial hot springs. Aside from SHF, the only other studied alkaline submarine hydrothermal vents are hosted in ultramafic rocks, rather than basalt, and are fueled by serpentinization, which creates high pH and millimolar concentrations of hydrogen and methane (Table 4). Prony hydrothermal field (PHF), located in New Caledonia is a shallow vent (16–47 m) where freshwater fluids influenced by serpentinization mix with seawater (Table 4; Quéméneur et al., 2014; Postec et al., 2015). The Lost City and Old City hydrothermal fields, also fueled by serpentinization, are much deeper (700–900 m and 3,100 m, respectively) and the source water for the vents is marine, rather than freshwater (Table 4; Kelley et al., 2005; Lecoeuvre et al., 2021). While elevated compared to seawater, the H_2_ and CH_4_ concentrations at SHF are in the micromolar range and therefore not as elevated as is found at serpentinite-hosted sites (Table 4). Therefore, high pH is the strongest geochemical similarity between SHF and serpentinite-hosted hydrothermal vents.

**TABLE 4:** Comparison of geology and geochemistry of submarine alkaline vents.

	Strýtan HF^1^	Prony HF^2,3^	Lost City HF^4^	Old city HF^5^
pH	10.2	10.6	11	7.88–8.18
Temp (C)	70	40	90	Not reported
(H_2_)	0.30–5.19 μM	6.4 mM	1–15 mM	Not reported
(CH_4_)	0.46–1.41 μM	8.0 mM	1–2 mM	Not reported
Water depth (m)	20–60	16–47	700–900	3,100
Host rock	Basalt	Ultramafic	Ultramafic	Ultramafic
Water source	Meteoric	Meteoric	Marine	Marine
Cone material	MgSiO_2_	CaCO_3_	CaCO_3_	CaCO_3_
Serpentinization	No	Yes	Yes	Yes

^1^Price et al. (2017). ^2^Postec et al. (2015). ^3^Quéméneur et al. (2014). ^4^Kelley et al. (2005). ^5^Lecoeuvre et al. (2021).

**FIGURE 5:**
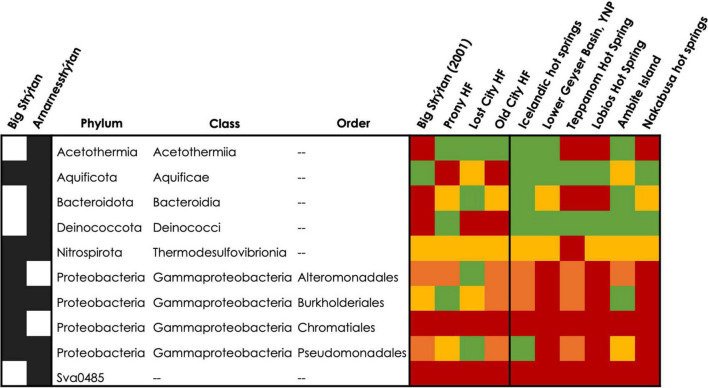
Detection of abundant taxa from SHF at other alkaline submarine hydrothermal vents and hot springs. Abundant ASVs (>5%) are listed at the class level, or order level for Proteobacteria. In the heatmap, the colors represent: red = not present, orange = phylum present (or class for Proteobacteria), yellow = class present (or order for Proteobacteria), and green = detected at a lower taxonomic level or the same level as in the current study. Additional details about these abundant ASV’s can be found in Tables 2, 3 and Supplementary Table 3.

Similarities between phylum through genus for the single observed Archeon and class through genus for Bacteria were included with the lowest common taxonomic level reported in Supplementary Table 3, with the closest matches highlighted in bright colors and higher (more distant) matches in faint colors. Commonalities between PHF and SHF include Acetothermia, *Thermus*, and Rhodocyclaceae (Gammaproteobacteria), as well as an unclassified bacterium, with 99% sequence similarity. Lost City and Old City hydrothermal fields shared Nitrosopumilaceae, Acetothermia, Thermodesulfovibrionia, Thiomicrospiraceaea, and various families belonging to the Bacteroidota and Gammaproteobacteria within SHF (Figure 5 and Supplementary Table 3). Notably, *Flavobacterium* and *Thermocrinis* abundant in SHF were absent in other alkaline submarine systems.

Our BLASTn results of abundant (>5%) ASVs indicate that the inhabitants of SHF are actually more similar to terrestrial hot spring samples than to marine sites (Tables 2, 3). Therefore, we additionally compared our diversity data to alkaline terrestrial hot springs (Table 5 and Figure 5 and Supplementary Table 3), such as Lower Geyser Basin, Yellowstone National Park, United States, Teppanom Hot Spring in Thailand, Lobios Hot Spring, Spain, Ambitle Island in Papua New Guinea, Nakabusa Hot Springs, Japan, and geothermal springs from southern Iceland. Notably, SHF has a higher pH than these sites (with the exception of other Icelandic sites) and a potentially high energy mixing zone where geothermal waters come into contact with oxygenated seawater (Table 5).

**TABLE 5:** Comparison geochemistry of SHF and terrestrial hot spring sites.

	Geographic location	pH	Temp °(C)
Strýtan HF^1,2^	Iceland	10.2	70
Icelandic hot springs^3^	Iceland	9–10	70–90
Lower geyser basin^4,5,6,7^	Yellowstone NP, United States	8–9.5	70–90
Teppanom hot spring^8^	Thailand	9	89
Lobios hot spring^9^	Spain	8.2	76
Ambitle Island^10^	Papua New Guinea	8.5	93.5
Nakabusa hot springs^11,12^	Japan	8.9	72–80

^1^This Study. ^2^Price et al. (2017). ^3^Tobler and Benning (2011). ^4^Reysenbach et al. (1994). ^5^Blank et al. (2002). ^6^Boomer et al. (2009). ^7^Meyer-Dombard et al. (2011). ^8^Purcell et al. (2007). ^9^López-López et al. (2015). ^10^Meyer-Dombard and Amend (2014). ^11^Nakagawa and Fukui (2003). ^12^Nishihara et al. (2018).

*Thermocrinis* and *Thermus* are two hallmark genera of terrestrial hot spring systems and were detected in most of the hot spring sites compared here (Figure 5 and Supplementary Table 3; Reysenbach et al., 1994; Blank et al., 2002; Nakagawa and Fukui, 2002; 2003; Purcell et al., 2007; Boomer et al., 2009; Meyer-Dombard et al., 2011; Meyer-Dombard and Amend, 2014; López-López et al., 2015; Nishihara et al., 2018; Martinez et al., 2019). However the exact species detected at SHF, *Thermocrinis albus* and *Thermus islandicus*, were only previously found in other hot springs in Iceland (Figure 5 and Supplementary Table 3; Marteinsson et al., 2001a; Tobler and Benning, 2011). Additional taxa that were found in terrestrial hot springs and SHF include the Nitrosopumilus, Acetothermia, Thermodesulfovibrionia, and Desulfobacteriota. Abundant taxa that are unique to SHF, compared to alkaline submarine and terrestrial geothermal systems, include Thermoanaerobaculaceae, Hydrogenispora, *Hydrogenophaga*, Sutterellaceae, Ca. *Alysiosphaera*, Ca. *Thiobios*, SAR324, and Sva0485 (Figure 5 and Supplementary Table 3).

## Conclusion

Strýtan Hydrothermal Field is a unique site, both geochemically and microbiologically. Close relatives to sequences found here have been described in submarine hydrothermal vents, terrestrial hot springs, Antarctica, the deep subsurface, and geothermal ground waters, suggesting it represents an amalgamation of various different environments and therefore a unique habitat. Community members from the vent fluids are mostly aerobic heterotrophic bacteria; however, within the chimneys we see taxa putatively capable of acetogenesis, sulfur-cycling, and hydrogen metabolism. Previous studies have hypothesized that alkaline vents, such as the ones described here could be a potential site for the origin of life on Earth (Russell, 2009; Russell et al., 2010). Additionally, the steep sodium gradient at SHF, which is not present at the other vents, could make it an even more likely analog for the cradle of life (Martin and Russell, 2007; Lane and Martin, 2012; Price et al., 2017). While the presence of O_2_ in seawater at SHF and other alkaline vents—and therefore a bioenergetically favorable oxygen gradient with reduced hydrothermal fluids—makes these sites inexact analogs for the early Earth, which was anoxic at the time of the origin of life (e.g., Farquhar et al., 2000; Johnson et al., 2014), this may actually position these systems as optimal analogs for habitable environments on other planets. In particular, both Mars (due to photochemistry and hydrogen escape) and Europa (due to radiolysis of surface ice followed by mixing with the subsurface ocean) possess now, and likely have for much of their history, O_2_ at concentrations that may be sufficient to support aerobic metabolisms (Hand et al., 2007; Lanza et al., 2016; Stamenković et al., 2018; Ward et al., 2019). Future metagenome-, metatranscriptome-, and culture-based work at SHF may therefore serve as an excellent natural laboratory to investigate the possibility of the origin and survival of life elsewhere in the solar system. Here we demonstrate that SHF is not only geochemically distinctive compared to other submarine hydrothermal vents, but also microbiologically unique. Future studies of the system may further help us to decipher the possible roles of both freshwater and marine alkaline hydrothermal systems in the origin of life.

## Data availability statement

The data presented in this study are deposited in the NCBI SRA repository, accession number PRJNA861241.

## Author contributions

KT, LW, WB, SM, RP, and DG contributed to the conception and design of the study. KT, LW, RP, DG, and SM participated in the sample collection and processing. KT, LW, ZK, AS, HP, RP, and WB contributed to the data analysis, curation, methodology, visualization, and validation. KT wrote the original draft. SM organized the field expedition and acquired the funding. All authors contributed to the interpretation, contributed to the review, editing, and approval of the final submitted version.
