# Supplementary material for: Microbial ecology of a shallow alkaline hydrothermal vent: Strýtan Hydrothermal Field, Eyjafördur, northern Iceland
Source: Front Microbiol. 2022 Nov 17;13:960335. doi: 10.3389/fmicb.2022.960335 (PMC9713835; doi:10.3389/fmicb.2022.960335)
Supplement: Supplementary file 1 [file Image_1.pdf]

Supplement to:

Microbial Ecology of a Shallow Alkaline Hydrothermal Vent: Strytan Hydrothermal Field, Eyjafjord, Northern Iceland

KI Twing, LM Ward, ZK Kane, A Sanders, R Price, HL Pendleton, D Giovannelli, WJ Brazelton, SE McGlynn

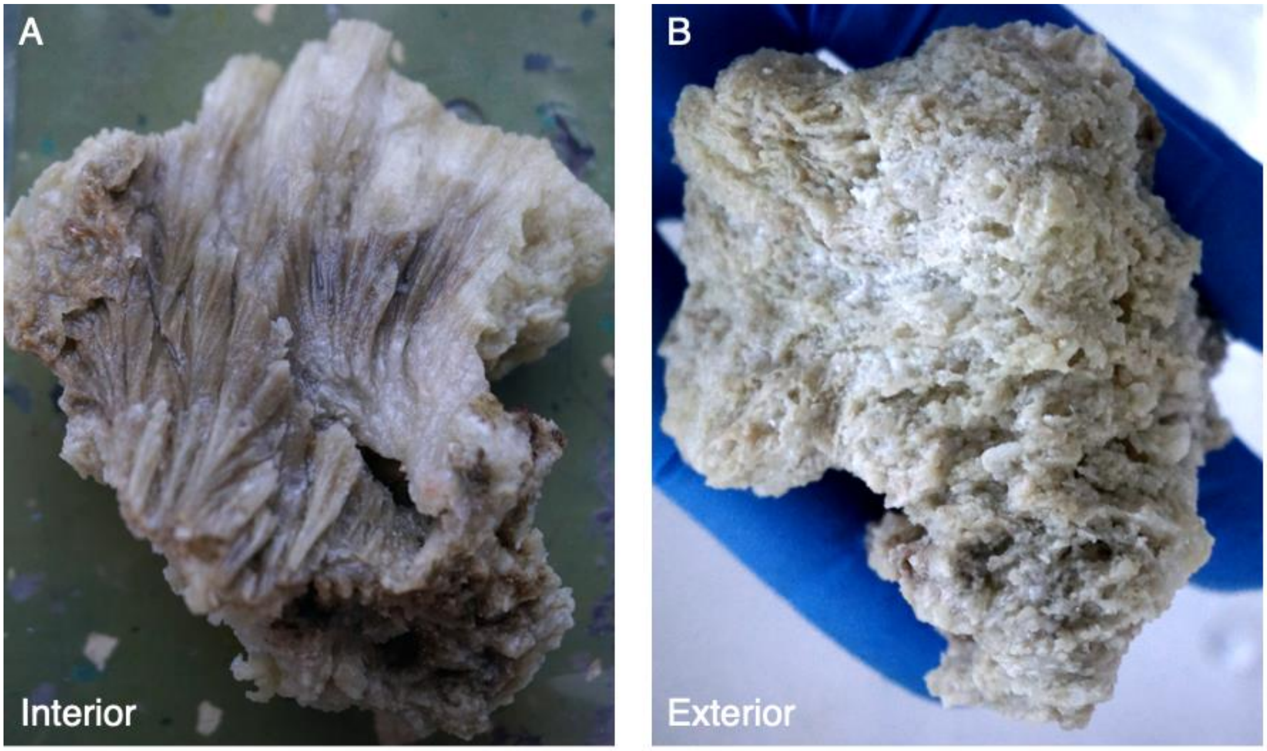

Figure S1 – Interior (A) and exterior (B) of Big Strytan chimney sample collected during Dive 7. Microbial samples ICEd054 and ICEd055 correspond to the interior and ICEd056 and ICEd057 correspond to the exterior (Table S1).
