# Supplementary material for: Microbial ecology of a shallow alkaline hydrothermal vent: Strýtan Hydrothermal Field, Eyjafördur, northern Iceland
Source: Front Microbiol. 2022 Nov 17;13:960335. doi: 10.3389/fmicb.2022.960335 (PMC9713835; doi:10.3389/fmicb.2022.960335)
Supplement: Supplementary file 2 [file Image_2.pdf]

Supplement to:

Microbial Ecology of a Shallow Alkaline Hydrothermal Vent: Strytan Hydrothermal Field, Eyjafjord, Northern Iceland

KI Twing, LM Ward, ZK Kane, A Sanders, R Price, HL Pendleton, D Giovannelli, WJ Brazelton, SE McGlynn

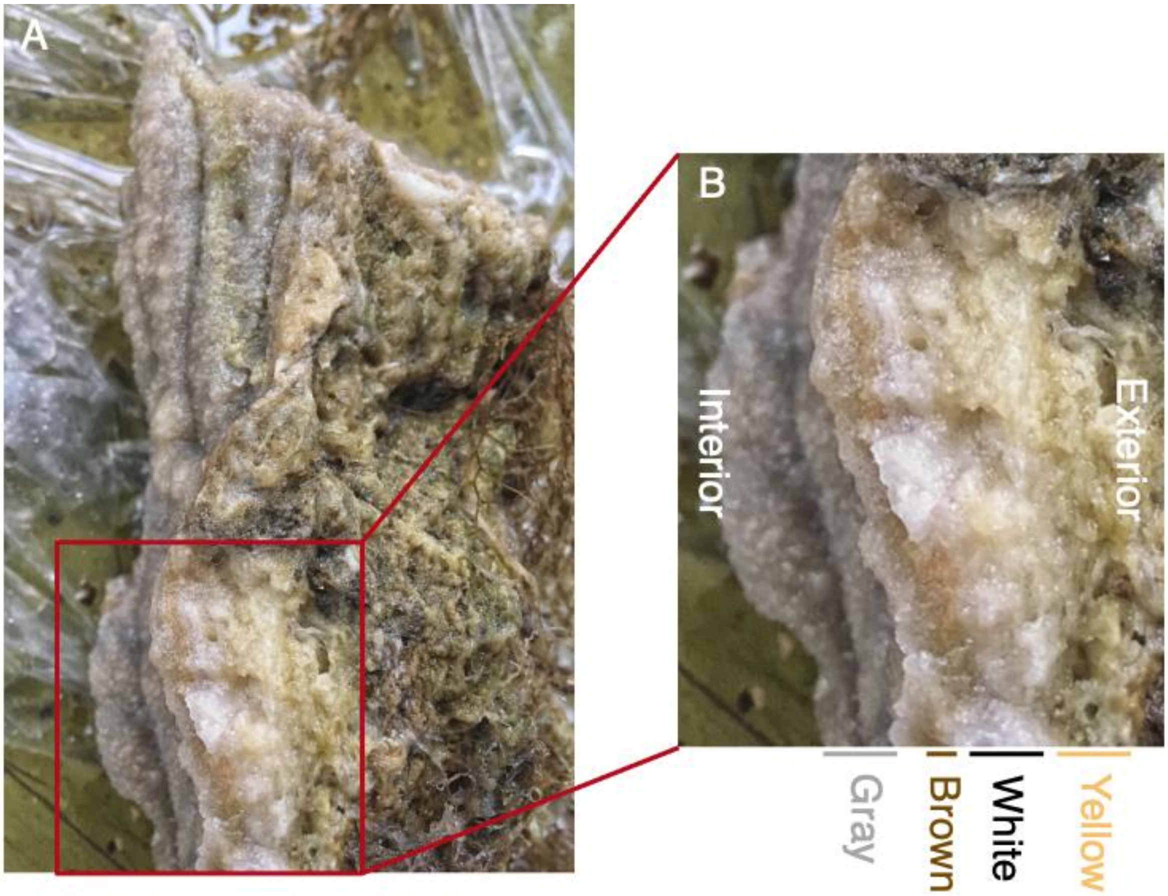

Figure S2 – Arnarnesstrýtan chimney sample (A) and close-up of color subsections (B) collected during Dive 12. The following microbial samples correspond to the following subsectioned layers: ICEd016 and ICEd017 to gray (interior), ICEd013 and ICEd014 to brown, ICEd019 and ICEd020 to white, and ICEd011 and ICEd012 to yellow (exterior) (Table S1).
